# Bioaccessibility, bioavailability and toxicity of commercially relevant iron- and chromium-based particles: in vitro studies with an inhalation perspective

**DOI:** 10.1186/1743-8977-7-23

**Published:** 2010-09-03

**Authors:** Yolanda Hedberg, Johanna Gustafsson, Hanna L Karlsson, Lennart Möller, Inger Odnevall Wallinder

**Affiliations:** 1 Div. Surface and Corrosion Science, Royal Institute of Technology (KTH), Drottning Kristinas väg 51, SE-100 44 Stockholm, Sweden; 2 Unit for Analytical Toxicology, Department of Biosciences and Nutrition, Novum, Karolinska Insitutet, SE-141 86 Huddinge, Stockholm, Sweden

## Abstract

**Background:**

Production of ferrochromium alloys (FeCr), master alloys for stainless steel manufacture, involves casting and crushing processes where particles inevitably become airborne and potentially inhaled. The aim of this study was to assess potential health hazards induced by inhalation of different well-characterized iron- and chromium-based particles, i.e. ferrochromium (FeCr), ferrosiliconchromium (FeSiCr), stainless steel (316L), iron (Fe), chromium (Cr), and chromium(III)oxide (Cr_2_O_3_), in different size fractions using in vitro methods. This was done by assessing the extent and speciation of released metals in synthetic biological medium and by analyzing particle reactivity and toxicity towards cultured human lung cells (A549).

**Results:**

The amount of released metals normalized to the particle surface area increased with decreasing particle size for all alloy particles, whereas the opposite situation was valid for particles of the pure metals. These effects were evident in artificial lysosomal fluid (ALF) of pH 4.5 containing complexing agents, but not in neutral or weakly alkaline biological media. Chromium, iron and nickel were released to very low extent from all alloy particles, and from particles of Cr due to the presence of a Cr(III)-rich protective surface oxide. Released elements were neither proportional to the bulk nor to the surface composition after the investigated 168 hours of exposure. Due to a surface oxide with less protective properties, significantly more iron was released from pure iron particles compared with the alloys. Cr was predominantly released as Cr(III) from all particles investigated and was strongly complexed by organic species of ALF. Cr_2_O_3 _particles showed hemolytic activity, but none of the alloy particles did. Fine-sized particles of stainless steel caused however DNA damage, measured with the comet assay after 4 h exposure. None of the particles revealed any significant cytotoxicity in terms of cell death after 24 h exposure.

**Conclusion:**

It is evident that particle and alloy characteristics such as particle size and surface composition are important aspects to consider when assessing particle toxicity and metal release from alloy particles compared to pure metal particles. Generated results clearly elucidate that neither the low released concentrations of metals primarily as a result of protective and poorly soluble surface oxides, nor non-bioavailable chromium complexes, nor the particles themselves of occupational relevance induced significant acute toxic response, with exception of DNA damage from stainless steel.

## Background

Assessment of respiratory effects induced by iron- and chromium-based particles are relevant for occupational exposure scenarios in industrial settings such as ferrochromium production sites. Ferrochromium alloys are produced from pyrometallurgical reduction processes of chromite ore. Dust and fume emissions generated during the smelting process are controlled by means of wet scrubbers, cyclones and bag filters of relatively high removal efficiency. However, non-controlled emissions take place both during the smelting and tapping process, as well as during crushing and sizing processes of the casted material into lumps and particles [1]. Handling and wind erosion of stockpiles of manufactured alloys will inevitably also result in airborne dust emissions that potentially may become inhaled. Today, the knowledge about potential respiratory effects induced by ferrochromium alloy particles is limited and often based on epidemiological investigations on poorly characterized particles [1-3] or on assumptions from toxicological findings relevant for individual alloy constituents such as chromium (Cr) and iron (Fe) [4]. Hexavalent chromium (Cr(VI)) is a known carcinogen (non-essential metal) via inhalation, and can for instance cause lung and sinonasal cancer in humans [5]. However, the international agency for research on cancer (IARC) states that there is inadequate evidence for any carcinogenicity of trivalent (Cr(III)) (essential metal) and metallic chromium [6]. Since hexavalent and trivalent chromium are linked in many production processes, potential toxicity induced by Cr(III) may be masked behind effects of Cr(VI), and their effects should therefore be investigated separately. Occupational studies on the carcinogenicity of Cr(III) exposure (e.g. ferrochromium industry and leather tannery workers) show no conclusive results and toxicological studies may therefore aid in understanding toxicity of various particle compositions and size fractions [6]. Reported occupational studies from stainless steel plants reveal however some lung accumulation of particles and respiratory symptoms at specific work places along the production line [3] and long term daily exposures to low concentration levels of dust and fumes containing Cr(III) generate enhanced respiratory symptoms, phlegm, shortness of breath and breathlessness on exertion [2]. No severe respiratory changes or lung cancer were however observed in any of these studies [7,8].

This study forms a part of a large research effort conducted at the Royal Institute of Technology (KTH), Sweden, the International Chromium Development Association (ICDA), France, and the Finnish Institute of Occupational Health (FIOH), Finland, investigating commercially relevant ferrochromium and ferrosiliconchromium alloy particles to enable a REACH-compliant chemical safety assessment of ferrochromium alloys [4]. In vitro bioaccessibility studies in synthetic biological fluids of relevance for the main human exposure routes; inhalation, oral (ingestion), and dermal (skin contact) in relation to detailed particle and surface analytical investigations have been conducted on particles of ferrochromium and ferrosiliconchromium alloys, globally relevant for the market [9,10]. In comparison, parallel studies have been conducted on pure iron and chromium metals, their oxides and stainless steel. From these and other studies [11-14], it is evident that the extent of metal release cannot be predicted from the nominal bulk or surface composition. Differences in surface reactivity and the importance of the chemical form of released metal species (important for bioavailability, see e.g. [15]) in relation to toxicity are evident. Furthermore, even though toxic effects of many metal containing particles seem to be related to soluble metal species, effects of the particle itself could not be excluded unless the mechanisms are fully understood [16,17].

The aim of this study was to assess potential health hazards induced by inhalation of different iron- and chromium-based particles. This was done by *i) *performing a careful characterization in terms of particle size and shape, size distribution, surface area and surface oxide composition, *ii) *assessing the extent and speciation of released metals (i.e. bioaccessibility and aspects of bioavailability) in synthetic biological medium, and *iii) *analyze particle reactivity and toxicity towards cultured human lung cells.

## Results

### Particle characterization

**Figure 1:**
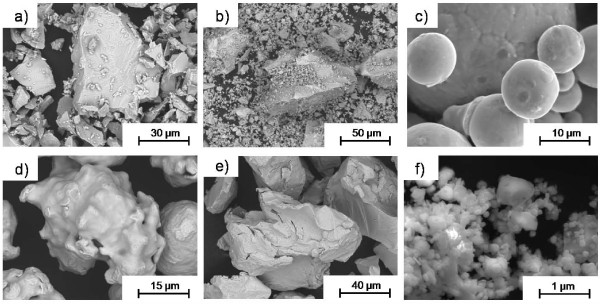
**Particle morphology of FeCr, FeSiCr, 316L, Fe, Cr, and Cr_2_O_3 _by means of BE-SEM**. SEM images of different magnification showing the morphology of the different particle types, with a) FeCr coarse (representative for FeCr fine and FeCr dust as well), b) FeSiCr fine (representative for FeSiCr coarse and FeSiCr dust as well), c) 316L coarse (representative for 316L fine as well), d) Fe coarse (as Fe fine), e) Cr coarse (as Cr fine), and f) Cr_2_O_3_.

Compiled information on particle characteristics in terms of particle shape and morphology (scanning electron microscopy, SEM), specific surface area (Brunauer-Elmer-Teller method, BET), and particle size distribution by means of laser diffraction (LD) in an aqueous medium of high ionic strength, PBS (phosphate buffered saline) is presented in Figure 1 and in Table 1 for all particles investigated (iron metal-Fe; chromium metal-Cr; ferrochromium-FeCr; FeCr dust; ferrosiliconchromium-FeSiCr; FeSiCr dust; stainless steel-316L; chromium(III)oxide-Cr_2_O_3_). More detailed information of coarser sized particles (based on BET-area) of Fe, Cr, FeCr, FeSiCr, coarse and fine particles of 316L and Cr_2_O_3 _is given elsewhere [9,10,18,19]. Particles of Cr, FeCr, FeSiCr, FeCr dust and FeSiCr dust revealed sharp edges and brittle visible cracks. These features were introduced during the crushing, sieving and re-crushing pre-process necessary to generate particles small enough to represent inhalation of respirable particles (90% of the number of fine particles were smaller than 5 μm (diameter in solution, including possible agglomerates), with the exception of fine chromium particles, see Table 1) but still have a chemical composition representative for the commercial products. The particle shapes of 316L particles were spherical and more irregular for particles of Fe. As expected, the specific surface area was significantly larger for the finer sized particles compared to the coarser sized particles, in particular evident for 316L, Fe, and Cr, Table 1. Despite the sieving process, SEM investigations still revealed the presence of some larger sized particles (> 20 μm).

**Table 1:** Particle characterization

			Cr	Fe	**Cr**_**2**_**O**_**3**_	FeCr	FeSiCr	FeCr dust	FeSiCr dust	316L
Coarse	Volume/diameter - μm	d_0.1_	57.5	37.1	-	6.9	3.4	47.0	8.8	11.7
		
		d_0.5_	104	101	-	22.4	22.8	297	41.9	22.5
		
		d_0.9_	170	181	-	53.7	121	769	107	41.4
	
	Number/diameter - μm	d_0.1_	1.1	0.74	-	0.94	0.64	0.8	0.74	7.4
		
		d_0.5_	1.5	1.0	-	1.5	0.98	1.2	1.1	11.3
		
		d_0.9_	2.8	1.9	-	3.7	2.1	2.4	2.7	20.3
	
	*BET-area*	*m^2^/g*	*0.04*	*0.05*	*-*	*0.19*	*0.51*	*0.06*	*0.23*	*0.07*

Fine	Volume/diameter - μm	d_0.1_	59.7	36.6	0.72	56.3	3.5	-	-	2.4
		
		d_0.5_	104	83.1	1.52	98.0	27.4	-	-	5.0
		
		d_0.9_	173	167	4.34	157	117	-	-	34.1
	
	Number/diameter - μm	d_0.1_	6.9	0.66	0.47	0.85	0.51	-	-	1.5
		
		d_0.5_	10.1	0.92	0.68	1.1	0.77	-	-	2.2
		
		d_0.9_	39.0	1.6	1.23	2.1	1.6	-	-	4.1
	
	*BET-area*	*m^2^/g*	*0.91*	*0.84*	*5.1*	*0.48*	*1.08*	*-*	*-*	*0.7*

Measured BET specific surface area [m^2^/g] (adsorbent: nitrogen, at five different partial pressures with a standard deviation of less than 1% - except for Fe coarse: 8%) and corresponding median particle diameters (d_0.5_) and the 10% (d_0.1_) and 90% (d_0.9_) size distribution cut-off points, presented as volume (mass) and numbers, determined from size distribution measurements in phosphate buffered saline (PBS) by means of laser diffraction.

The specific BET-surface area (surface area per unit mass) increased according the following sequence;

*Cr-coarse ≈ Fe-coarse ≈ FeCr-dust ≈ 316L-coarse < FeCr-coarse < FeSiCr-dust < < FeCr-fine ≈ FeSiCr-coarse < 316L-fine < Fe-fine < Cr-fine < FeSiCr-fine < < Cr_2_O_3_*.

According to size distribution measurements, Table 1, agglomeration occurred for all fine sized particles, in particular pronounced for the fine sized particles of Cr and Cr_2_O_3_. Tabulated size distribution numbers correspond to primary particles and/or agglomerates formed in solution, a distribution that may be different at dry conditions.

According to surface compositional analysis by means of x-ray photoelectron spectroscopy, XPS, chromium, iron, and silicon were the main components of the outermost surface (< 5 nm) oxide on particles of FeCr, FeSiCr, FeCr-dust and FeSiCr-dust, in non-proportional relations to their corresponding bulk compositions, Table 2. The relative mass surface content of oxidized silicon was significantly higher for dust particles of FeCr (Si_ox_/(Fe_ox_+Cr_ox_+Si_ox_): 0.46 ± 0.04) compared to both coarse (0.07 ± 0.03) and fine sized particles (0.08) at the same time as the relative amount of chromium was lower (Cr_ox_/(Fe_ox_+Cr_ox_+Si_ox_): 0.41 ± 0.02-dust; 0.65-fine; 0.70 ± 0.01-coarse). A similar effect, although less pronounced, was observed for FeSiCr with a slightly higher relative mass surface content of oxidized silicon for its dust particles (Si_ox_/(Fe_ox_+Cr_ox_+Si_ox_):0.41 ± 0.01 compared to 0.37 ± 0.06), and a corresponding lower oxidized Cr content (0.42 ± 0.03 compared to 0.50 ± 0.03). XPS findings imply slightly thinner surface oxides on dust particles on both alloys compared with their corresponding surface oxides on coarse and fine particles.

**Table 2:** Nominal bulk composition (wt%) of alloys and pure metals

	Cr	Fe	C	Si	Ni	Mn	Mo	S	Cu	V
**FeCr**	67.0	25.0	7.1	1.1	0.4	0.2	-	-	-	0.1

**FeSiCr**	35.8	21.7	0.05	42.5	-	-	-	-	-	-

**316L**	16.8	68.9	0.03	0.5	10.3	1.4	2.1	0.01	-	-

**Cr**	> 99.76	0.14	0.007	0.04	-	-	-	0.003	-	-

**Fe**	-	> 99.96	0.002	-	-	-	-	-	-	-

Ferrochromium (FeCr), ferrosiliconchromium (FeSiCr), stainless steel (316L), chromium metal (Cr), and iron metal (Fe) - supplier information.

Unexpectedly, fine metal particles of Cr revealed oxidized iron in the outermost surface oxide (Fe_ox_/(Fe_ox_+Cr_ox_): 0.19), but not for the coarser sized particles of Cr.

The surface oxide of fine particles of 316L (Cr_ox_/(Fe_ox_+Cr_ox_): 0.26) was more enriched in oxidized chromium compared to the coarse particles (0.18). Oxidized manganese was detected in the surface oxide of both particle fractions. The results imply higher manganese content in the surface oxide on the finer sized particles. However due to the significant overlap between the Ni-LMM Auger and the Mn 2p peaks, no quantitative estimate of the manganese surface content was made.

### Bioaccessibility in a synthetic lung fluid

**Figure 2:**
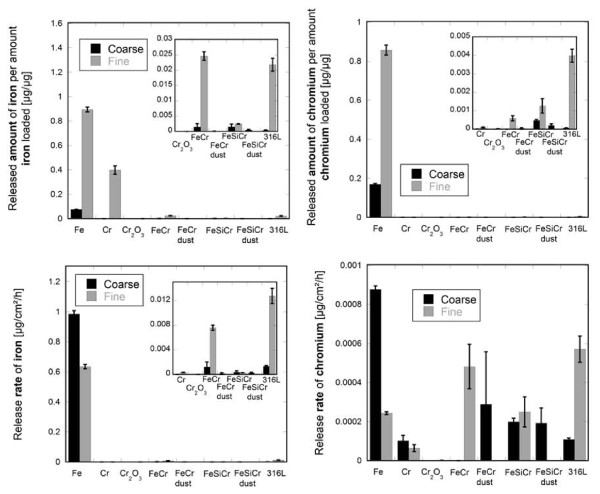
**Release of iron and chromium from alloys, pure metals and chromium oxide in ALF**. Amounts of iron and chromium released per amount of iron and chromium in particles loaded (μg/μg) (top), and corresponding release rates (μg/cm^2^/h) (bottom) from coarse and fine sized particles (based on BET area) of alloys (FeCr, FeSiCr, 316L), alloy dust (FeCr dust, FeSiCr dust), pure metals (Fe, Cr), and chromium(III)oxide (Cr_2_O_3_) immersed in artificial lysosomal fluid (pH 4.5) for 168 h (1 week). Error bars represent the standard deviation of triplicate samples. Inset graphs are of higher magnification.

Released amounts of iron and chromium per amount of particles loaded and corresponding release rates normalized to the particle BET-surface area and immersion period in artificial lysosomal fluid (ALF) of pH 4.5, simulating inflammatory conditions at 37°C, are presented for all particles investigated in Figure 2 after 168 hours (1 week) of immersion.

Except for particles of Cr_2_O_3 _(no measurable release of iron) and FeSiCr-fine particles (about the same amount of iron and chromium is released), significantly more iron was released compared with chromium, also for particles of pure fine-sized Cr metal (> 99.76 wt% chromium). Compared with chromium, the released amount of iron was approximately 20 times higher for 316L-fine particles, 16 times higher for FeCr-fine particles, and 6 times higher for Cr-fine particles.

The release of iron from all alloys and alloy-dust particles was significantly lower compared with particles of Fe metal (Figure 2). After one week of immersion in ALF, particles of Fe-fine and Fe-coarse were completely (≈1 μgFe/μgFe), and to a large extent (0.076 μgFe/μgFe, i.e. 7.6%), dissolved/released. For the alloys, only a very small amount of iron was released (< 0.022 μgFe/μgFe - 316L-fine). Similar or lower amounts of iron were released from dust particles of FeCr and FeSiCr compared with coarse and fine particles of FeCr and FeSiCr.

The released amount of chromium from particles of Cr metal was similar or lower compared to findings for particles of the alloys, despite more oxidized chromium present in the surface oxide of pure chromium compared with the alloys. In addition, when normalized to the BET surface area, it was evident that the release rate of chromium after one week of immersion in ALF was lower for chromium metal particles (0.000064 μg/cm^2^/h-Cr-fine) compared to all alloy particles (FeCr-fine: 0.0005 μg/cm^2^/h; FeSiCr-fine: 0.00025 μg/cm^2^/h; 316L-fine: 0.0006 μg/cm^2^/h), and the Fe metal particles (Fe-fine: 0.00024 μg/cm^2^/h). The highest release rate of chromium (0.0009 μg/cm^2^/h) was determined for Fe-coarse metal particles (> 99.96 wt% iron) and the lowest rate for particles of Cr_2_O_3 _(0.0000024 μg/cm^2^/h - 0.00003 μgCr/μgCr). Both when normalized to the specific surface area (Figure 2, bottom) or to the bulk iron and chromium content of the particles (figure 2, top), similar or lower amounts of both chromium and iron were released from dust particles of FeCr and FeSiCr compared with coarse and fine particles of FeCr and FeSiCr.

Nickel was released to a very low extent from alloy particles of FeCr-coarse (< 0.0002 μg/cm^2^/h, below limit of detection), FeSiCr-coarse (< 0.0002 μg/cm^2^/h, below limit of detection) and 316L-fine (< 0.0004 μg/cm^2^/h) after one week of immersion in ALF [9,10]. For the alloy particles, when only considering the iron, chromium, and nickel release, the remaining mass fraction of particles after one week of exposure to ALF corresponded to 99.96% (FeCr - coarse), 99.35% (FeCr - fine), 99.95% (FeSiCr - coarse), 99.90% (FeSiCr - fine), 99.98% (316L - coarse), 98.40% (316L - fine), 99.996% (FeCr dust), and 99.98% (FeSiCr dust).

When comparing metal release rates, which take into account differences in surface area, it is evident that all fine alloy particles investigated revealed higher rates of both chromium and iron compared to their coarser particle sizes, whereas the opposite situation was true for the pure metals. These findings were statistically significant for both chromium and iron released from FeCr and 316L (p < 0.01) and for chromium released from FeSiCr (p < 0.001) but not for iron released from FeSiCr (p > 0.05).

### Bioaccessibility in other biological fluids

**Figure 3:**
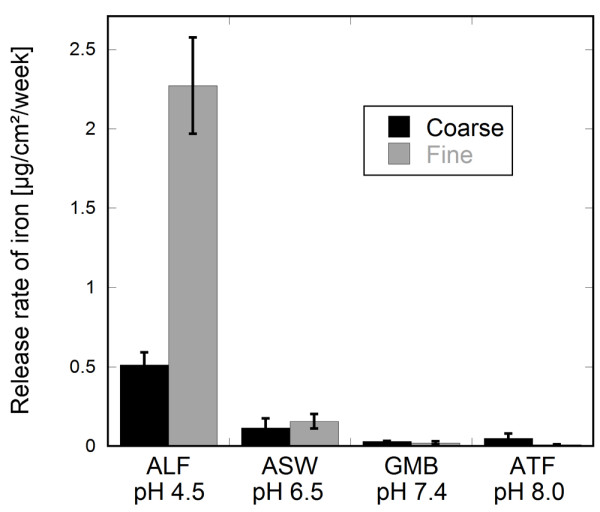
**Influence of biological media and pH on the release rate of iron from stainless steel particles**. Release rates of iron (μg/cm^2^/week) from particles of 316L (coarse and fine) immersed in artificial lysosomal fluid (ALF), artificial sweat (ASW), Gamble's solution (GMB) and artificial tear fluid (ATF) for 168 h (1 week). Error bars represent the standard deviation of triplicate samples.

To investigate if these findings were also evident in other biological fluids, release rates of iron from fine and coarse particles of 316L are presented in Figure 3 after one week of immersion in each fluid. Higher release rates of iron from finer compared to coarse-sized particles were only statistically significant (p < 0.001) in the acidic medium of ALF (pH 4.5) of complex chemical composition, and not in the less complex, pH neutral and weakly alkaline media of artificial sweat, ASW (pH 6.5), Gamble's solution, GMB (pH 7.4) and artificial tear fluid, ATF (pH 8.0). The chemical composition of each medium is presented in Table 3. Similar trends were seen for determined release rates of chromium.

**Table 3:** Chemical composition (g/L) of artificial biological fluids

Chemicals	ALF pH 4.5	ASW pH 6.5	GMB pH 7.2	ATF pH 8.0
MgCl_2_	0.050	-	0.095	-
NaCl	3.21	5.0	6.019	6.78
KCl	-	-	0.298	1.38
Na_2_HPO_4_	0.071	-	0.126	-
Na_2_SO_4_	0.039	-	0.063	-
CaCl_2_·2H_2_O	0.128	-	0.368	0.084
C_2_H_3_O_2_Na	-	-	0.574	-
NaHCO_3_	-	-	2.604	2.18
C_6_H_5_Na_3_O_7_·2H_2_O	0.077	-	0.097	-
KH_2_PO_4_	-	-	-	-
NaOH	6.00	-	-	-
C_6_H_8_O_7_	20.8	-	-	-
H_2_NCH_2_COOH	0.059	-	-	-
(NH_2_)_2_CO	-	1.0	-	-
CH_3_CHOHCO_2_H	-	1.0	-	-
C_4_H_4_O_6_Na_2_·2H_2_O	0.090	-	-	-
C_3_H_5_NaO_3_	0.085	-	-	-
C_3_H_3_O_3_Na	0.086	-	-	-

Artificial lysosomal fluid (ALF), pH 4.5, artificial sweat (ASW) (according to EN1811), pH 6.5, Gamble's solution (GMB), pH 7.4, and artificial tear fluid (ATF), pH 8.0. Detailed information is given in [9,10].

**Figure 4:**
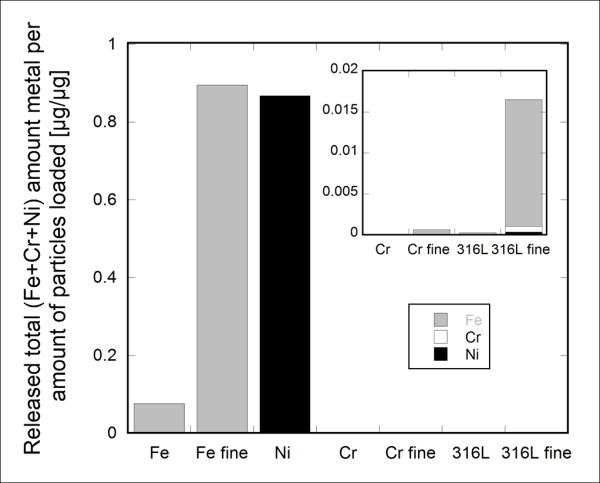
**Total metal release from alloys and pure metal particles in ALF**. Amount of total iron, chromium, and nickel released per amount of particles loaded (μg/μg) from coarse and fine particles of 316L, pure Fe, pure Cr, and pure Ni when immersed in artificial lysosomal fluid for 168 h (1 week). Error bars represent the standard deviation of triplicate samples. The inset graph is of higher magnification.

The fact that alloys and pure metals behave differently from a metal release perspective is illustrated in Figure 4 for fine and coarse particles of 316L and the pure metals Cr, Ni and Fe, immersed for one week in ALF. Significantly larger amounts of both iron and nickel were released from the pure metals compared with the alloy of both size fractions. Very low amounts of chromium were released from both the pure chromium metal and the alloys. Kinetic information (relatively high initial release rate (< 24 hours), strongly decreasing with time) on the release of alloy constituents from stainless steel as massive and particles, respectively, in ALF is presented elsewhere [13,18,20].

### Chemical speciation of released chromium

**Figure 5:**
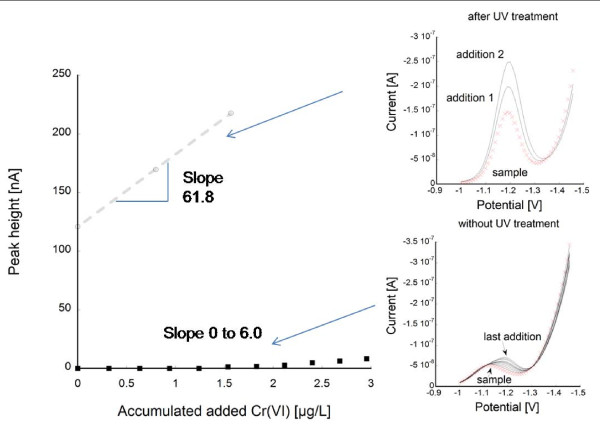
**Speciation measurements of chromium in ALF**. Known addition of Cr(VI) to assess the complexation capacity of ALF to chromium before and after UV treatment (destroying organic species) by means of voltammetry (DPAdCSV). The signal of free chromium (labile chromium) is measured (peak height) while the total chromium content in the solution is known, and hence the complexed chromium can be obtained.

Chemical speciation measurements of released chromium by means of stripping voltammetry (DPAdCSV) on selected samples (Cr-coarse, FeCr-coarse, and 316L-coarse) after one week of exposure in ALF revealed chromium to be released as Cr(III). No evidence of any Cr(VI) in solution was perceived (< 0.01 μg/L). To assess the complexation capacity of ALF for Cr, standard addition of Cr(VI) was conducted. This is illustrated in Figure 5 for a given ALF solution with a chromium concentration of 15.8 μg/L. The gradual addition of Cr(VI) (in total 1.5 μg/L) revealed a complexing capacity of the solution of 17.3 μg/L when considering strong ligands only, as concluded from the lack of Cr(VI) in solution (no Cr peak). Further addition of Cr(VI) into the same solution revealed a slight increase of the Cr peak, indicative of Cr(VI) ions in solution. A significantly lower slope of the added Cr(VI) to peak height signal compared with the slope obtained from the same solution being UV treated to destroy complexing organic ligands clearly revealed a high complexing capacity also to weak ligands. The results show that organic ligands of the ALF medium have a high capacity to complex released chromium, at least if released at ppb-concentrations (μg/L).

### Toxicological testing of relevance for an inhalation scenario

Due to the fact that fine-sized particles have a higher probability of reaching the conducting airways or the alveoli, fine-sized particles of the alloys (FeCr, FeSiCr, 316L) and the pure metals (Fe, Cr) were subjected to different toxicological investigations of relevance for an inhalation scenario. Cr_2_O_3 _and NiO were investigated for comparative reasons, and single experiments were performed on larger dust particles (FeCr dust and FeSiCr dust) to exclude that these did not show unexpectedly higher toxicity than the fine sized fraction.

**Figure 6:**
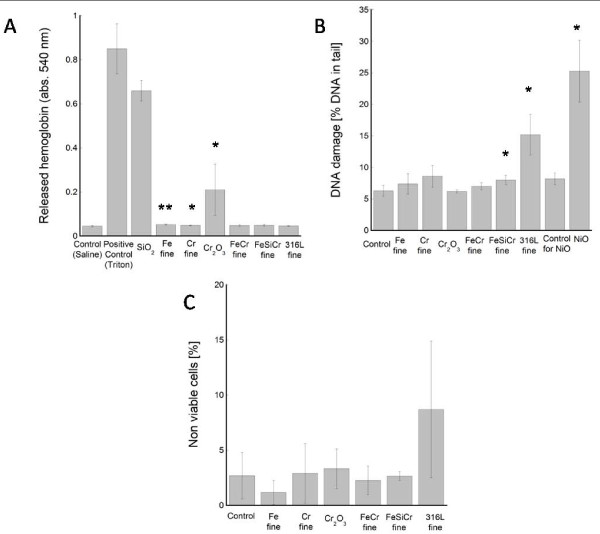
***In vitro *toxiciological testing**. 6A shows the release of hemoglobin after erythrocytes were incubated with the different particle suspensions at 2.67 mg/mL. Presented numbers are mean values of three independent experiments. The absorbance was measured in duplicate wells (n = 6). DNA damage (after 4 h) and cytotoxicity (after 24 h) following exposure of A549 cells are displayed in 6B and 6C (mean values of three independent experiments). Asterisks (*) indicate statistically significant increase compared to controls with p-values of 0.05 (*) or 0.01 (**), respectively.

Surface reactivity was analyzed as hemolysis of red blood cells (erythrocytes) in three different concentrations, 0.67, 1.33 and 2.67 mg/mL. Figure 6A shows the release of hemoglobin, measured as absorbance intensity at 540 nm, for the dose 2.67 mg/mL. Each particle suspension was measured against a saline control and 1% Triton-X 100 as positive control causing total hemolysis. In addition, SiO_2 _particles were used as a positive particle control. As seen in the figure, Cr_2_O_3 _particles caused hemolysis whereas none of the alloy particles or dust particles did. Calculated as % hemolysis, the Cr_2_O_3 _particles caused 1.4%, 3.1% and 22.5% hemolysis (mean value of three independent experiments) in the different doses. This can be compared to the SiO_2 _particles causing 43.1%, 62.0% and 78.5% in the different doses.

The alkaline version of the comet assay, measuring mainly DNA single strand breaks and alkaline labile sites, was used to assess DNA damage after exposure of A549 human lung cells to a concentration of 40 μg/cm^2 ^(80 μg/mL) for 4 hours. Significantly higher amount of DNA damage was seen following exposure to 316L (16%, p < 0.05) when compared to the control (6%), see Figure 6B. A minor but statistical significantly effect (p < 0.05) was also observed for FeSiCr fine particles (8.0%). NiO nanoparticles, tested for comparison, showed approximately 4 times higher amount of DNA damage (25.3% tail) when compared to controls. None of the other particles caused any significant DNA damage.

Cytotoxic effects in terms of cell death were investigated after exposure of A549 human lung cells for 24 hours to 40 μg/cm^2 ^(80 μg/mL) particles. A slight, although non-significant, increase in cell death was observed after exposure to 316L particles. None of the other particles showed any effect (Figure 6C).

## Discussion

To understand which properties that drive the toxicity of various particles, it is crucial to enable a screening of a large number of particles by using *in vitro *methods. Surface oxide characteristics, such as composition, stability and thickness, of metal containing particles change with decreasing particle size and are of decisive importance for the metal release process, and most probably also the toxicological response. This study (after 168 hours of exposure) and previous kinetic findings at different time points and in different media clearly demonstrate that the extent of alloy constituents released from alloy particles into a given medium is neither proportional to the bulk nor to the surface composition [9,10,13,14,18,20,21]. This emphasizes the fact that metal release processes are the combined result of electrochemical, chemical and wear phenomena.

Previous kinetic studies of coarse FeCr particles in ALF and other synthetic body fluids [9], of coarse FeCr, Fe, Cr, coarse and fine 316L, and Cr_2_O_3 _particles in artificial sweat and tear fluid [10], coarse and fine 316L particles in ALF [18], and of different grades of massive stainless steel in ALF [13,20,21] and other media [14,21], all show a relatively high initial metal release rate, strongly decreasing with time due to the development of a very stable mixed iron(II)oxide and chromium(III)oxide passive layer (enriched in chromium compared with the bulk composition) for all iron- and chromium-based alloy particles. Relative comparisons between different alloy or pure metal particles and different particle sizes are valid at all different time points investigated in these studies (up to one month) when using average release rates (as in this study). Only one time point was therefore selected to make a relative comparison between coarse and fine particles of alloys and pure metals. However, it should be underlined that observed differences presented in this study are only valid for the exposure time of 168 hours. Presented rates are average values for the entire exposure period, including the relatively high initial metal release during the first hours. Momentary metal release rates (e.g. the metal release between the fourth and seventh day of exposure) would be significantly lower. The metal release mechanism is governed by electrochemical processes (corrosion, diffusion-controlled for passive metals as in this study), physical processes (e.g. wear), and chemical processes (chemical dissolution of the oxide layer). In this study, the latter may be the most important due to the dominant presence of complexing agents in ALF. This weakens covalent bonds in the outermost metal oxide and hydroxide layer and results in the release of metal-ligand complexes [22]. Based on unpublished data, electrochemical processes were believed to be of minor importance for the coarse and fine particles of 316L.

Findings in this study for alloy particles (316L) clearly illustrate increased metal release rates (per surface area) of alloy constituents with decreased particle size in concordance to changes in surface oxide composition assessed by means of XPS. These effects were pronounced in the acidic synthetic medium of ALF (pH 4.5) simulating an inflammatory condition, but less evident for more pH-neutral or weakly alkaline media of less complexity, (e.g. Gamble's solution, GMB), see Figure 3. On-going studies by the authors indicate that unique components of ALF, e.g. the high citric acid content, in combination to structural differences between the particles of different size, is a main reason for significantly higher metal release rates observed for fine sized (surface area: 0.7 m^2^/g) compared with coarser (0.07 m^2^/g) particles of 316L.

Increasing metal release rates (per surface area) with decreasing particle size could not be confirmed for the pure metals particles, actually displaying the opposite behavior. A similar effect has also been observed for micron and nano sized copper metal particles in PBS after different exposure time periods [23]. The reason is most probably combined effects of particle agglomeration (more commonly occurring for small-sized particles), confirmed by the light diffraction measurements for small particles, and/or a thermodynamic equilibrium shift between released metal ionic species in solution and the metal surface in the closed experimental system (smaller particles are releasing more dissolved metal into solution, which then decreases further dissolution due to thermodynamic considerations). Similar effects have been observed in other biological systems [10,13,18,20,24]. Particle agglomeration has previously been shown to decrease the initial release of copper from non-sonicated copper metal nanoparticles compared with sonicated particles [23].

All Fe-Cr based metal particles investigated in this study have a high bulk content of chromium (FeCr-67 wt%, FeSiCr-36 wt% and 316L-17 wt%). This content exceeds by a margin the content of 10.5-13 wt% required to form Cr(III)-rich surface oxides with high barrier properties for oxidation (corrosion) [25-27] and metal release [9,10,13,18,20]. Such passive surface oxides are generally not present on metal containing airborne particulate matter (PM), which usually has a high content of organic matter [28].

As a result of the chromium-enrichment of the surface oxide, metals were released at very low concentrations (sub-μg/L) from all investigated particles in this study. Particles with the largest content of chromium, Cr and Cr_2_O_3_, revealed the lowest extent of chromium release (per surface area) as a result of the highest barrier properties of their surface oxides. The very low release rates of chromium is further evident based on the finding that coarse-sized particles of Fe metal with a poorly protective surface oxide actually displayed the highest release of chromium per surface area of all particles investigated. For further perspective on the extent of chromium release, rain water-concrete interactions result in orders of magnitude higher concentrations of released chromium [29].

Despite a high nickel bulk content of 316L (10.3 wt%), no nickel was observed in the surface oxide. Very low levels of nickel were released for both particle size fractions [9,10]. According to literature findings, nickel is present in an alloy surface layer beneath the passive surface oxide [30,31]. As long as the surface oxide on stainless steel remains intact, it acts as an efficient barrier also for nickel release. Locally and intermittently occurring corrosion events can though change this effect [32]. Significantly higher amounts of nickel were released from pure nickel metal particles (Ni) as a result of significantly less efficient barrier properties of the surface oxide compared to the passive chromium-rich oxide on stainless steel. Similar effects were also evident for pure Fe, forming surface oxides with relatively poor barrier properties, Figure 4.

Further evidence for the importance of surface oxide properties is the fact that fine-sized particles of Fe metal with a relatively poorly protective surface oxide of high solubility were almost completely dissolved in ALF after one week of exposure, whereas only a small fraction of the amount of particles loaded (< 1.5% - fine; < 0.04% - coarse) was dissolved/released for the alloys with highly protective and poorly soluble chromium-rich surface oxides. Released metals are often denoted as bioaccessible metals, i.e. metals that may be, or become, bioavailable and absorbed by human cells and induce toxicity. However, the bioavailability is strongly related to the chemical speciation of released metals, which in turn depends on the chemistry of the medium of interest and its complexation capacity for released metals [15]. Chemical speciation findings by means of stripping voltammetry revealed chromium to be released as Cr(III) without any traces of Cr(VI) for all particles investigated. In addition, organic components of the ALF medium displayed a high capacity to predominantly form strongly bonded complexes and to some extent weakly bonded complexes with chromium. These complexes have usually a low bioavailability [15]. An even higher complexation capacity is expected for the cell medium used for the toxicological investigations due to even higher content of organic species and presence of serum. Whether complexation of released metals with different ligands in a given medium is advantageous or not from a metal release and toxicological perspective can be discussed. Even though non-bioavailable and non-toxic complexes often are formed, this complexation process can trigger the extent of released metals [33]. Depending on the protective character and solubility of the surface oxide, a small or large portion of the particles is dissolved/released into solution, thereby possibly changing the surface area, the particle reactivity, surface characteristics and potential concomitant toxic effects induced, for instance by the smaller particle size obtained. At released metal concentrations, exceeding the complexation capacity of the medium, free metal ions may exist in solution and promote toxicity.

Even though released ionic species can be of high importance for toxicity induced by particles, it is well known within particle toxicology that certain particles with low solubility can be toxic due to a reactive particle surface [34,35]. As a measure of particle reactivity, the hemolytic assay was used in the present study. This method measures the ability of the particles to destroy the integrity of red blood cells and has been shown to be a good predictor for the *in vivo *inflammatory potential of various particles such as different silica particles [36], alumina (Al_2_O_3_) and NiO nanoparticles [37]. None of the fine alloy particles tested in this study showed however any hemolytic activity indicating low particle reactivity and possibly low inflammatory potential. Hemolytic activity was however seen for Cr_2_O_3 _particles, but the reason for this is unclear. These particles had however the largest surface area (5.1 m^2^/g) of all particles tested, which may be part of the explanation. Preliminary surface charge measurements of these particles show that sonication, a procedure conducted prior to the toxicological tests, could significantly increase the surface charge when compared with both non-sonicated and other particles investigated. This could hence be an important part of the increased hemolytic activity observed for these particles and needs to be further investigated.

To analyze toxicity of the fine-sized particles to human lung cells, DNA damage was assessed after 4 h exposure and cell death after 24 h exposure to a concentration of 40 μg/cm^2 ^particles. This concentration and approximately these time points have been used previously to distinguish between particles of high toxicity such as Cu and CuO nanoparticles [23,38] and particles with lower toxicity including various iron oxide particles [39,40]. The results showed DNA-damaging effects of fine-sized 316L particles but not for the other particles, except from a minor increase by FeSiCr. Previous findings imply that free trivalent chromium ions slowly can interact in a dose-dependent manner to DNA and spontaneously cause mutations [41]. However, released chromium is a non-likely explanation for observations made in this study since the release was very low and ions were rapidly complexed with ligands in solution. Neither is the release of nickel a possible explanation due to its very low release rates. It should however be noted that all metal release measurements in this study were performed in particle suspensions of a non-sonicated simple synthetic biological medium, whereas the toxicological investigations were conducted with serum-supplemented and sonicated cell medium, a worst case from a metal release perspective. Still, a more probable reason for the DNA damage may be related to the direct particle-cell interaction. Our results also indicated cell death after 24 h of exposure to 316L particles, although this effect was not statistical significant due to high variations between different experiments. Difficulties and variations in the assessment of cytotoxicity of nano-sized (40 nm) 316L particles are also evident from a recent interlaboratory exercise where contradictory results (no and high toxicity, respectively) were presented [42].

The relatively low toxicity of the particles investigated in this study is in agreement with literature findings. Except for respiratory symptoms related to sustained irritation at total dust levels of 2.5 mg/m^3 ^observed for plant workers at stainless steel production sites crushing and sintering ferrochromium alloys, no other acute effects have, so far, been observed for humans [2,3]. The rather low toxicity is also in concordance with a recent systemic inhalation toxicity study on rats, showing no adverse effects after 28 days of repeated daily inhalation of 316L particles (fine-the same as in this study) at aerosol dose levels of up to 1.0 mg/L [43]. Potential carcinogenicity of specific chromium compounds generally relates to their solubility in biological fluids and the ability of its particles or dissolved ionic species to enter target cells [6]. Neither chromium metal nor trivalent chromium compounds examined in this study are water-soluble, as evidenced from very low released concentrations of chromium in this study and other studies [44]. Since ferro-chromium alloys have Cr(III)-rich surface oxides, read-across possibilities with trivalent chromium compounds and chromium metal are suggested by on-going risk assessments to assess potential adverse effects [4]. The restricted release of iron and other minor alloy constituents are in the same risk assessment considered minor and non-toxic.

Inhalation studies with rats exposed to trivalent chromium oxide aerosols in concentrations ranging from 4.4 to 44 mg/m^3 ^did not result in any systemic toxic effects [11]. The same study revealed however some accumulation of alveolar macrophages and slight inflammation in alveolar epithelium at all dose levels. Epidemiological studies at stainless steel production plants show similar results with no, or low toxicity induced by inhalation of ferrochromium dust particles [4,6,44,45].

**Figure 7:**
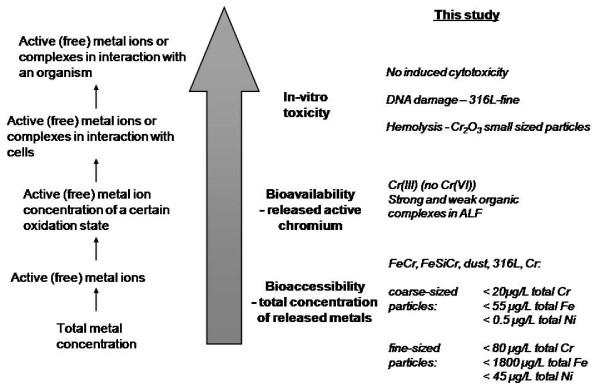
**Schematic summary**. Compilation of results generated on metal release (bioaccessibility), chemical speciation and complexation of released chromium (important factors for bioavailability) in ALF, and toxicity induced by fine and coarse sized particles of iron- and chromium-based particles.

A schematic compilation of key results generated from *in vitro *studies of iron- and chromium-based particles assessing their bioaccessible pool of iron and chromium released in synthetic biological fluid, the chemical speciation (oxidation state and complexation), important for the bioavailability of released chromium, and their toxicological response on cytotoxicity, DNA damage and hemolysis of erythrocytes, is shown in Figure 7. This interdisciplinary study reveals no or low toxicity induced by iron- and chromium-based, potentially inhalable, metal and alloy particles relevant for occupational scenarios. Parallel *in vitro *bioaccessibility and toxicological studies are important to understand fundamental mechanisms, and should be combined with in-depth particle characterizations with a surface perspective.

## Conclusions

Health hazards induced by potential inhalation of iron- and chromium-based particles of relevance for an occupational exposure scenario were assessed in this study by investigating release of metals and their chemical speciation in artificial lung fluids as well as particle reactivity (hemolytic activity) and damage to cultured human lung cells. The study included particles of ferrrochromium alloys and their dust particles (high-carbon ferrochromium, ferrosiliconchromium), stainless steel (AISI 316L), pure metals (Fe, Cr) and chromium(III)oxide of different size distributions and surface areas. The following main conclusions were drawn:

• The extent of released metals, normalized to the particle surface area, increased with decreasing particle size (increasing surface area) for the alloys, but decreased for particles of the pure metals. This effect was evident in the complex artificial lysosomal fluid (pH 4.5), but not in the pH neutral or weakly alkaline biological media investigated (pH 6.5-8.0).

• Surface oxide composition and properties such as electrochemical passivity govern the extent of released metals. However, no correlation exists with the surface or the bulk composition.

• Chromium was released in very low concentrations (sub-μg/L) as trivalent chromium species from the alloys, pure Cr and Cr_2_O_3 _particles, and was strongly complexed primarily by organic species in the artificial lysosomal fluid. This complexation capacity is expected to be even higher at realistic cell medium conditions.

• High particle reactivity, measured as hemolytic activity towards erythrocytes, was only observed for particles of Cr_2_O_3_. The reason is unclear but these particles had the largest surface area, which may be part of the explanation.

• Particles of stainless steel induced DNA damage. The damage was likely not due to metal ionic species investigated but rather to particle/cell interactions.

• None of the particles caused cell death in the dose and time-point tested, although stainless steel particles showed a non-statistical significant increase.

• It is clear that particle characteristics such as particle size, surface oxide composition and barrier properties are important aspects to consider when assessing both toxicity and release of metals from particles. Bulk compositional data is not sufficient for such assessment. Further essential aspects to consider include the chemical speciation of released metals (oxidation state and complexation) in different media.

## Methods

### Materials

Alloy particles of a high-carbon ferrochromium alloy, FeCr, and ferrosiliconchromium, FeSiCr, of commercial relevance were supplied by Vargön Alloys AB, Sweden and Kazchrome, Republic of Kazakhstan, respectively [9]. These alloys are used as master alloys for further production of more advanced steel alloys. As the commercial products are different solid forms (lumps) and particle sizes, the materials were pre-treated (crushed, sieved, and re-crushed) to generate particles with a size distribution less than 60 μm. Dust particles of FeCr and FeSiCr were created during standardized dustiness tests using DIN 55992-1 [46]. Coarse (< 45 μm) and fine (< 4 μm) particles of stainless steel (gas-atomized, AISI 316L, used for powder metallurgy molding) were supplied by Arcam AB, Sweden, and Sandvik Osprey Limited, UK, respectively [18]. The nominal bulk composition of particles of FeCr, FeSiCr, and 316L is given in Table 2 based on supplier information. For comparative reasons, particles of Fe metal (99.96 wt%, Höganäs, Sweden), Cr metal (99.76 wt%, Delachaux, France), and chromium(III)oxide (99.1 wt%, Lanxess Deutschland GmbH, Germany) were investigated in parallel.

Particles of FeCr, FeSiCr, Fe, and Cr were further milled and sieved (20 μm sieve) by Elektrowerk Weisweiler GmbH, Germany, to obtain a smaller size fraction (< 20 μm). Particles sized less than 100 μm (aerodynamic diameter) are referred to as inhalable, particles less than 11 μm are defined as thoracic particles able to pass the larynx, and particles sized less than 5 μm are defined as respirable particles, able to reach the alveolar region of the deep lung (ISO 7708). It should be noticed that the aerodynamic diameter is larger than the physical diameter, with a relationship of approximately aerodynamical diameter = physical diameter * √density_particle _(see e.g. [47]). In this study, the particle size distribution was measured in solution, accounting for agglomeration and hence particle cut-off points are larger compared with physical diameters measured in dry air.

### Particle characterization

The *specific surface area *(m^2^/g) per mass unit was determined by means of BET-analysis (adsorption of nitrogen at cryogenic condition) using a Micromeritics Gemini V instrument. Nitrogen adsorption measurements were performed at five different partial pressures (p/p_0 _0.10-0.25) with a standard deviation between replicas of less than 1% (except for Fe coarse particles: 8%). The adsorbent used was nitrogen, with a cross-sectional diameter of 0.162 nm^2 ^as input parameter. The sample was dried with nitrogen and flushed in a sample tube for 30 minutes at 150°C. After the measurement, several criteria (e.g. comparison to references) were verified, and if not sufficient, the measurement was repeated. The total surface area was about or larger than 1 m^2 ^to ensure accurate measurements using nitrogen gas. The measurement was conducted by Kanthal AB, Sweden.

Triplicate measurements of *particle size distribution *in solution (PBS, phosphate buffered saline, 8.77 g/L NaCl, 1.28 g/L Na_2_HPO_4 _and 1.36 g/L KH_2_PO_4_, pH 7.2) were conducted by means of laser diffraction using a Malvern Mastersizer 2000 equipment with a Hydro SM dispersion unit. Refractive indexes of Fe (2.81), Cr (3.51), Fe_3_O_4 _(2.42), Cr_2_O_3 _(2.5), and water (1.33) (since it was the solvent of the test medium), were used as input parameters applying standard operational conditions.

*Particle shape and morphology *were investigated by means of scanning electron microscopy (SEM), with a PHILIPS XL30ESEM instrument, and a field emission gun scanning electron microscopy (FEG-SEM) using a LEO 1530 instrument and a Gemini column.

*Compositional analysis of surface oxides *was performed by means of x-ray photoelectron spectroscopy, XPS (UltraDLD spectrometer from Kratos Analytical, Manchester, UK) using a monochromatic Al x-ray source (150 W) on areas approximately sized 700 × 300 μm. Wide spectra were run to detect elements present in the outermost surface oxide (information depth of a few nanometers) at five different locations, and detailed high resolution spectra (20 eV pass energy) were acquired for the main compositional elements. Experimental details are given in [9].

### Bioaccessibility studies

Metal release studies were conducted by immersing a specific particle loading (100 mg/L: 5 mg/50 mL) in artificial lysosomal fluid (ALF) of pH 4.5 (37°C) for exposure of 168 hours (1 week). A loading of 200 mg/L was used in the case of the fine 316L particles [18]. The loading of 100 or 200 mg/L has previously been shown to be low enough to avoid additional particle agglomeration in solution due to a too high loading [19]. The loading of 100 mg/L is furthermore recommended by the OECD transformation/dissolution protocol for aquatic acute tests [48]. ALF simulates in a simple way intracellular inflammatory conditions in lung cells following phagocytosis [49]. The composition of ALF is given in Table 3. The pH (4.5 ± 0.1) was adjusted by using 1.7 mL 50% NaOH to 1L solution. Chemical compositions of artificial sweat (ASW, pH 6.5), Gamble's fluid (GMB, pH 7.2), and artificial tear fluid (ATF, pH 8.0) are given in Table 3, and detailed information for these fluids given in [9,10]. All immersion studies were conducted at dark conditions in acid-cleaned polymethylpentene (PMP) Nalgene^® ^jars using Merck mini incubators standing on a shaking table with bi-linear shaking (30 cycles per minute, inclination 12°). Time-dependent studies were conducted using triplicate particle samples and one blank sample (without any particles). After exposure, the upper part of the test solution was poured into tubes and the particles were separated from the solution by centrifugation at 704 rcf (relative centrifugal force) for 10 minutes. This separation technique has previously been shown to be the most suitable procedure for bioaccessibility studies of similar metal particles [19]. To ensure sufficient separation of particles in the supernatant sampled from the centrifuged test medium, dynamic light scattering (Malvern Zetasizer nano ZS instrument) was performed. The supernatant, free of particles, was then poured into 25 mL polyethylene storage flasks and acidified to a pH less than 2 prior to metal analysis.

Selected samples of coarse sized Cr, FeCr, and 316L particles were immersed for 24 hours into the ALF solution to assess the chemical speciation (oxidation state and medium complexation capacity) of chromium. Non-acidified samples for these speciation analyses were immediately frozen. In order to avoid any metal contamination, all test vessels and experimental equipment were acid-cleaned with pure 10% HNO_3 _for at least 24 hours and rinsed at least four times with ultra pure water (18.2 MΩ/cm). All experimental equipment and vessels were dried in ambient laboratory air before use. Experimental details are given in [9,10].

### Metal analysis and speciation

Total iron, chromium, and nickel (coarse FeCr and FeSiCr, coarse and fine 316L particles) concentrations were analyzed by means of graphite furnace atomic absorption spectroscopy, GF-AAS (Perkin Elmer AAnalyst 800), or flame atomic absorption spectroscopy (AAS) for higher (mg/L) concentrations (Fe and Ni particles). All measurements are based on three replicate readings of each sample, and quality control samples of known concentration were analyzed consecutively. Calibration was done with at least three standards of known concentration, e.g. 50, 100, and 500 μg/L for iron. All results presented are based on measured metal concentrations in the supernatants (particles separated) with the contribution from blank reference samples (matrix effects), if any, subtracted. The limits of detection (based on 3 times the standard deviation of blank samples) were 1, 1, and 0.5 μg/L for iron, chromium, and nickel, respectively (GF-AAS); and 0.1 and 0.5 mg/L for iron and nickel, respectively (AAS).

Concentrations of active Cr(VI) and total chromium were determined by means of differential pulse adsorptive cathodic stripping voltammetry (DPAdCSV) using a Metrohm 797 VA Computrace (with a hanging drop mercury electrode working electrode, an Ag/AgCl sat. KCl reference electrode and Pt auxiliary electrode) instrument and Metrohm 705 UV digester (high pressure mercury lamp, 500 W, 90°C). Calibration was conducted individually for each sample by standard addition of Cr(VI) (sufficient to double the peak height). The detection limit was 0.04 μg/L for both total chromium and Cr(VI). All blank concentrations measured were significantly below the limit of detection. More detailed information on the methodology is given in [9].

### Cell culture and particle preparation for toxicity studies

Cells from the human alveolar type II-like epithelial cell line, A549 (originally obtained from the American Type Culture Collection, ATCC) were grown and exposed to particles in Dulbecco's Minimal Essential Medium (DMEM) supplemented with 10% heat inactivated foetal bovine serum (FBS), 100 U/mL penicillin-streptomycin, and 1 mM sodium pyruvate in a humidified atmosphere at 37°C and 5% CO_2_.

For the cytotoxicity assay, 0.08 million cells were seeded in wells of a 24-well plate (Becton Dickinson, Franklin Lakes, US) and grown for 24 h to obtain a 50% covering layer. After the additional exposure of particles for 24 h, a more than 90% confluent layer was obtained. In the comet assay 0.16 million cells were seeded in the wells and grown for 24 h to obtain a more than 90% confluent layer.

As a worst case inhalation scenario, the finer sized particles of FeCr, FeCr-dust, FeSiCr, FeSiCr-dust, Cr, Fe, and Cr_2_O_3 _were subjected for the toxicological testing. Before particle exposure of the cells, the particles were suspended in supplemented DMEM to a concentration of 1 mg/mL. The suspensions were vortexed for 20 s and sonicated using a probe (approximate output of 14 W in a 2 mL suspension) for 2 × 20 s with a 20 s break in between, to minimize particle agglomeration, and further diluted to 80 μg/mL (equal to 40 μg/cm^2 ^in the well plate) in 37°C sterile DMEM medium. Control cells were exposed to DMEM medium.

### Cytotoxicity

The cells were exposed to 40 μg/cm^2 ^(80 μg/mL) of particles for 24 h. After exposure, the cells were mixed with trypan blue, incubated for 3 min and the percentage of stained cells counted in a Bürker chamber, as a measure of cytotoxicity. The detailed experimental procedure is presented elsewhere [39]. A minimum of 100 cells was counted for each particle type in three separate experiments.

### DNA damage

The cells were exposed to 40 μg/cm^2 ^(80 μg/mL) of particles for 4 h. To investigate DNA damage, mainly single strand breaks (SSB), alkali-abile sites (ALS) and double strand breaks (DSB), the alkaline version of the comet assay (single cell gel electrophoresis) was performed, previously described in [50]. The electrophoresis was however performed at ~17 V in a Comet Electrophoresis tank (SCIE-PLAS, Comet -20) where the negatively charged DNA-fragments migrate out from the nucleus, creating a "comet" with a tail. Fixation and then staining of DNA with ethidium bromide (1 μg/mL in Tris acetate-EDTA (TAE)) was performed the next day and the results were evaluated by computerized image analysis with a fluorescence microscope (Olympus BH2 with a 20x-apochromatic objective) using the program Komet 4.0 (Kinetic Imaging Ltd.). For each sample, 35 cells were examined in duplicates (i.e. 70 cells) and a mean value of the DNA damage as % tail of the comets for three independent experiments was calculated.

### Hemolysis of erythrocytes

Fresh venous blood, from healthy blood donors (Blood Donor Center, Karolinska University Hospital, Stockholm), was collected in 10 mL EDTA tubes. The samples were gently mixed by inversion, added onto histopaque, centrifuged at 400 g (1370 rpm) for 30 min (20°C) and erythrocytes were collected. The cells were washed 3 times with PBS and then suspended in saline. 100 μL of suspended erythrocytes were mixed with 200 μL of particles in saline solution, pure saline or 0.1% Triton (negative and positive controls, respectively). The particles were prior to exposure of the erythrocytes suspended in saline to a concentration of 4 mg/mL, vortexed for 20 s and then sonicated in an ultrasonic bath for 15 min, to minimize agglomeration. The particles were then further diluted to 2 and 1 mg/mL (final concentration with erythrocytes; 2.67, 1.33 and 0.67 mg/mL). All exposures were conducted at dark conditions on a shaking table for 30 min (mixed every 10 min) and then centrifuged for 5 min at 10,000 rpm (15°C). Hemoglobin levels of the supernatant were determined from the optical density (OD), a measure of lysed erythrocytes, by using a microplate scanning spectrophotometer (PowerWave x, Bio-Tek Instruments, Inc. USA) at 540 nm (reference 620 nm) in two separate wells of the well plate. Three independent experiments were conducted for each particle. The % hemolysis was then calculated as presented in [37].

### Statistical data evaluation

Un-paired two-tailed Student's *t*-test with unequal variance was used for statistical analysis of all bioaccessibility and toxicity results.

## Competing interests

The authors declare that they have no competing interests.

## Authors' contributions

YH was involved in the experimental design, carried out part of the bioaccessibility studies and particle characterization, and drafted the manuscript. JG conducted the toxicity assays, was involved in the interpretation of the toxicity results and in the manuscript preparation. HLK was involved in the experimental design, interpretation of the toxicity results, and the final manuscript preparation. LM worked with the final version of the manuscript and was supervisor for the toxicological experiments. IOW organized the study and collaboration, designed the study, conducted the XPS analysis, and was involved in final manuscript preparation. All authors have approved the final manuscript.
